# Multilingual hope speech detection in English and Dravidian languages

**DOI:** 10.1007/s41060-022-00341-0

**Published:** 2022-07-10

**Authors:** Bharathi Raja Chakravarthi

**Affiliations:** Insight SFI Research Centre for Data Analytics, National University of Ireland Galway, Galway, Ireland

**Keywords:** Hope speech, Equality, Diversity, Inclusion, Multilingual, Dravidian Languages

## Abstract

Recent work on language technology has aimed to identify negative language such as hate speech and cyberbullying as well as improve offensive language detection to mediate social media platforms. Most of these systems rely on using machine learning models along with the labelled dataset. Such models have succeeded in identifying negativity and removing it from the platform deleting it. However, recently, more research has been conducted on the improvement of freedom of speech on social media. Instead of deleting supposedly offensive speech, we developed a multilingual dataset to identify hope speech in the comments and promote positivity. This paper presents a multilingual hope speech dataset that promotes equality, diversity and inclusion (EDI) in English, Tamil, Malayalam and Kannada. It was collected to promote positivity and ensure EDI in language technology. Our dataset is unique, as it contains data collected from the LGBTQIA+ community, persons with disabilities and women in science, engineering, technology and management (STEM). We also report our benchmark system results in various machine learning models. We experimented on the Hope Speech dataset for Equality, Diversity and Inclusion (HopeEDI) using different state-of-the-art machine learning models and deep learning models to create benchmark systems.

## Introduction

Recently, equality, diversity and inclusion (EDI) has attracted widespread attention with a focus on the protected classes of gender and race. It started as early as 1960, but it is only now that the interpretation of diversity has broadened to include other demographics such as the lesbian, gay, bisexual, transgender, queer/questioning (one’s sexual or gender identity), intersex, and asexual/aromantic/agender (LGBTQIA+) community, women in the fields of science, engineering, technology and management (STEM), and persons with disabilities. [1]. Inclusion refers to making an individual feel like they are a part of a group or organisation, both in terms of the formal and informal environment [2, 3]. Another essential part of this wheel is bias. People have both conscious and unconscious biases, which lead to explicit and implicit stereotyping, respectively. To avoid bias, much training has been provided to school students [4], employees and various levels [5]. However, it is only very recently that artificial intelligence (AI) researchers have started looking at biases, especially gender bias [6]. Language technologies in AI are expected to have a growing influence over our lives in the internet era. Nevertheless, from the perspective of language technologies research, the EDI for minority LGBTQIA+ or marginalised populations has not been considered with great urgency or importance compared to other topics or areas. It is important that the language technologies developed consider the inclusion of all communities for social integration.

Online social media platforms such as Facebook, Twitter and YouTube have encouraged millions of people to express themselves and share their opinions. These platforms also provide a medium for many marginalised people to look for support online [7–9]. The emergence of the infectious disease COVID-19 led to the exposure of the entire population to the disease without specific pharmacological treatment; the exponential levels of infection has deeply affected countries across the world, and the pandemic forced public places to remain closed temporarily [10]. Several areas have been affected worldwide, and the fear of losing loved ones caused even basic necessities such as schools, hospitals and mental health care centres to remain closed [11]. As a consequence, people were forced to look at online forums for their informational and emotional needs. In some areas and for some people, online social networking has been the only means of ensuring social connectedness and seeking social support during the COVID-19 pandemic [12].

Online social networking delivers a platform for network individuals to be in the know and to be known, both of which are more significant with more prominent social integration. Social integration is essential for the overall well-being of every individual, but most importantly vulnerable individuals who are more prone to social exclusion. A sense of belonging and community is an essential aspect of people’s mental health, which influences both psychological and physical well-being [13]. The importance of social inclusion in the online lives of marginalised populations, such as women in the fields of STEM, people who belong to the LGBTQIA+ community, racial minorities or people with disabilities, has been studied, and it has been proven that the online life of vulnerable individuals produces a significant impact on their mental health [14–16]. However, the contents of social media comments or posts may be negative, hateful, offensive or abusive since there is no mediating authority.

Comments and posts on social media have been analysed to find and stop the spread of negativity using methods such as hate speech detection [17], offensive language identification [18–20] and abusive language detection [21]. However, according to [22], technologies developed for the detection of abusive language do not consider the potential biases of the dataset that they are trained on. The systematic racial bias in the datasets causes abusive language detection to be biased, and this may result in discrimination against one group over another. This will have a negative impact on minorities or marginalised people. As language is a major part of communication, it should be inclusive. A large internet community that uses language technology has a direct impact on people across the globe. We should turn our attention towards spreading positivity instead of curbing an individual’s freedom of speech by removing negative comments. However, hope speech detection should be done alongside hate speech detection. Otherwise, hope speech detection by itself may lead to bias while perpetrators of negative and harmful comments continue to act wildly on the web.

Therefore, in our research, we focused on hope speech. Hope is commonly associated with the promise, potential, support, reassurance, suggestions or inspiration provided to participants by their peers during periods of illness, stress, loneliness and depression [23]. Psychologists, sociologists and social workers from the Association of Hope have concluded that hope can also be a useful tool for saving people from suicide or self-harm [24]. The ’Hope Speech’ delivered by gay rights activist Harvey Milk on the steps of the San Francisco City Hall during a mass rally to celebrate California Gay Freedom Day on 25 June 1978^1^ inspired millions to demand rights that ensure EDI [25]. Recently, [26] analysed how to use hope speech from social media texts to diffuse tensions between two nuclear powered nations (India and Pakistan) and support marginalised Rohingya refugees [27]. They experimented with detecting hope versus non-hope. However, to the best of our knowledge, no prior work has explored hope speech for women in STEM, LGBTQIA+ individuals, racial minorities or people with disabilities in general.

Moreover, although people from various linguistic backgrounds are getting exposed to online social media language, English remains at the centre of ongoing trends in language technology research. Recently, some research studies have been conducted on high-resourced languages such as Arabic, German, Hindi and Italian. However, such studies usually use monolingual corpora and do not examine code-switched textual data. Code-switching is a phenomenon where the individual switches between two or more languages in a single utterance [28]. We have introduced a dataset for hope speech identification not only in English but also in the under-resourced code-switched Tamil (ISO 639-3: tam), Malayalam (ISO 639-3: mal) and Kannada (ISO 639-3: kan) languages.

- We have proposed to encourage hope speech rather than take away an individual’s freedom of speech by detecting and removing a negative comment.
- We applied the schema to create a multilingual hope speech dataset for EDI. This is a new large-scale dataset of English, Tamil (code-mixed) and Malayalam (code-mixed) YouTube comments with high-quality annotation of the target.
- We performed an experiment on Hope Speech dataset for Equality, Diversity and Inclusion (HopeEDI) using different state-of-the-art machine learning and deep learning models to create benchmark systems.

## Related works

When it comes to crawling social media data, there are many works on YouTube mining [29, 30], which are mainly focused on exploiting user comments. [31] performed opinion mining and a trend analysis on YouTube comments. The researchers conducted an analysis of the sentiments to identify their trends, seasonality and forecasts, and it was found that user sentiments are well correlated with the influence of real-world events. [32] conducted a systematic study on opinion mining by targeting YouTube comments. The authors developed a comment corpus containing 35K manually labelled data for modelling the opinion polarity of the comments based on tree kernel models. [33] and [34] collected comments from YouTube and created a manually annotated corpus for the sentiment analysis of the under-resourced Tamil and Malayalam languages.

Methods to mitigate gender bias in natural language processing (NLP) have been extensively studied for the English language [35]. Some studies have investigated gender bias beyond the English language using machine translation to French [36] and other languages [37]. [38] studied the gender and dialect bias in automatically generated captions on YouTube. Technologies for abusive language [39, 40], hate speech [17, 41] and offensive language detection [42–44] are being developed and applied without considering the potential biases [22, 45, 46]. However, current gender debiasing methods in NLP are not sufficient to debias other issues related to EDI in the end-to-end systems of many language technology applications; this causes unrest and escalates the issues with EDI besides leading to greater inequality on digital platforms [47].

The use of counter-narratives (i.e. informed textual responses) is another strategy that has received the attention of researchers recently [48, 49]. A counter-narrative approach was proposed to weigh the right to freedom of speech and avoid over-blocking. [50] created and released a dataset for counterspeech using comments from YouTube. However, the core idea of directly intervening with textual responses escalates hostility even though it is advantageous for the writer to understand why their comment or post has been deleted or blocked and then favourably change the discourse and attitudes presented in their comments. Thus, we directed our attention to finding positive information such as hope and encouraging such activities.

Recently, a work by [26] and [27] analysed how to use hope speech from a social media text to diffuse tension between two nuclear powered nations (India and Pakistan) and support minority Rohingya refugees. However, the authors’ definition of hope was only confined to diffusing tensions and preventing violence. It did not take into account other perspectives on hope and EDI. The authors did not provide more information such as the inter-annotator agreement, diversity among annotators and details about the dataset. The dataset is not publicly available for research. It was created in English, Hindi and other languages related known to the Rohingyas. Our work differs from the previous works in that we have defined hope speech for EDI and introduced a dataset for English, Tamil and Malayalam on the EDI of it. To the best of our knowledge, this was the first work to create a dataset for EDI in Tamil and Malayalam, which are under-resourced languages.

## Hope speech

Hope is an upbeat state of mind based on a desire for positive outcomes in one’s life or the world at large, and it is both present and future-oriented [23]. Inspirational talks about how people deal with and overcome adversity may also provide hope. Hope speech instills optimism and resilience, which have a beneficial impact on many parts of life, including [51] college [52] and other factors that put us at risk [53]. For our problem, we defined hope speech as ’YouTube comments/posts that offer support, reassurance, suggestions, inspiration and insight’.

The notion that one may uncover and become motivated to use routes to their desired goals is reflected in hope speech. Our approach sought to shift the dominant mindset away from a focus on discrimination, loneliness or the negative aspects of life and towards a focus on promoting confidence, offering support and creating positive characteristics based on individual remarks. Thus, we instructed annotators that if a comment or post meets the following conditions, then it should be annotated as hope speech.

- The comment contains inspiration provided to participants by their peers and others and/or offers support, reassurance, suggestions and insight.
- The comment promotes well-being and satisfaction (past), joy, sensual pleasures and happiness (present).
- The comment triggers constructive cognition about the future—optimism, hope and faith.
- The comment contains an expression of love, courage, interpersonal skill, aesthetic sensibility, perseverance, forgiveness, tolerance, future-mindedness, praise for talents and wisdom.
- The comment promotes the values of EDI.
- The comment brings out a survival story of gay, lesbian or transgender individuals, women in science or a COVID-19 survivor.
- The comment talks about fairness in the industry (e.g. [I do not think banning all apps is right; we should ban only the apps which are not safe]).
- The comment explicitly talks about a hopeful future (e.g. [We will survive these things]).
- The comment explicitly talks about and says no to division in any form.

Non-hope speech includes comments that do not bring positivity, such as the following:

- The comment uses racially, ethnically, sexually or nationally motivated slurs.
- The comment produces hate towards a minority group.
- The comment is highly prejudiced and attacks people without thinking about the consequences.
- The comment does not inspire hope in the reader’s mind.

Non-hope speech is different from hate speech. Some examples are provided below.

- **’How is that the same thing???’** This is non-hope speech, but it is not hate speech.—explanation
- **’Society says don’t assume, but they assume to anyways’** This is non-hope speech, but it is not hate speech.—explanation

A hate speech or offensive language detection dataset is not available for code-mixed Tamil and code-mixed Malayalam, and it does not take into account LGBTQIA+ people, women in STEM or other minority or under-represented groups. Thus, we cannot use the existing hate speech or offensive language detection datasets to detect hope or non-hope for EDI of minorities.

## Dataset construction

We concentrated on gathering information from YouTube comments on social media,^2^ which is the most widely used platform in the world for commenting on and publicly expressing opinions about topics or videos. We did not use comments from LGBTQIA+ people’s personal coming out stories since they contained personal information. For English, we gathered information on recent EDI themes such as women in STEM, LGBTQIA+ concerns, COVID-19, Black Lives Matter, the United Kingdom (UK) versus China, the United States of America (USA) and Australia versus China. The information was collected from recordings of individuals from English-speaking nations like Australia, Canada, Ireland, the United Kingdom, the United States of America and New Zealand.

For Tamil and Malayalam, we gathered data from India on recent themes such as LGBTQIA+ concerns, COVID-19, women in STEM, the Indo-China war and Dravidian affairs. India is a country that is multilingual and multiracial. In terms of linguistics, India is split into three major language families: Dravidian, Indo-Aryan and Tibeto-Burman. The ongoing Indo-China border conflict has sparked online bigotry towards persons with East-Asian characteristics despite the fact that they are Indians from the North East. Similarly, in Tamil Nadu, the National Education Policy, which calls for the adoption of Sanskrit or Hindi, has exacerbated concerns about the linguistic autonomy of Dravidian languages. We used the YouTube comment scraper^3^ to collect comments. From November 2019 to June 2020, we gathered data on the aforementioned subjects. We believe that the statistics we have shared will help to reduce animosity and promote optimism. Our dataset was created as a multilingual resource to enable cross-lingual research and analysis. It includes hope speeches in English, Tamil and Malayalam, among other languages.

### Code-mixing

When a speaker employs two or more languages in a single speech, it is known as code-mixing. It is prevalent in the social media discourse of multilingual speakers. Code-mixing has long been connected with a lack of formal or informal linguistic expertise. It is, nevertheless, common in user-generated social media material according to studies. In a multilingual country like India, code-mixing is quite a frequent occurrence [54–57]. Our Tamil and Malayalam datasets are code-mixed since our data was collected from YouTube. In our corpus, we found all three forms of code-mixing, including tag, inter-sentential and intra-sentential. Our corpus also includes code-mixing between Latin and native scripts.

### Ethical concerns

Data collected from social media is extremely sensitive, especially when it concerns minorities such as the LGBTQIA+ community or women. By eliminating personal information from the dataset, such as names but not celebrity names, we have taken great care to reduce the danger of the data revealing an individual’s identity. However, in order to investigate EDI, we needed to keep track of the information on race, gender, sexual orientation, ethnicity and philosophical views. The annotators only viewed anonymised postings and promised not to contact the author of a remark. Only researchers who agree to follow ethical norms will be given access to the dataset for research purposes. We opted not to ask the annotator for racial information after a lengthy debate with our local EDI committee members.^4^ Due to recent events, the EDI committee was strongly against the collection of racial information based on the belief that it would split people according to their racial origin. Thus, we recorded only the nationality of the annotators.

**Table 1:** Annotators

Language		English	Tamil	Malayalam
Gender	Male	4	2	2
	Female	5	3	5
	Non-binary	2	1	0
Higher Education	Undergraduate	1	0	0
	Graduate	4	4	5
	Postgraduate	6	2	2
Nationality		Ireland, UK, USA, Australia	India, Sri Lanka	India
Total		11	6	7

### Annotation set-up

After the data collection phase, we cleaned the data using *Langdetect*^5^ to identify the language of the comments and removed comments that were not in the specified languages. However, owing to code-mixing at various levels, comments in other languages became unintentionally included in the cleaned corpus of the Tamil and Malayalam comments. Finally, based on our description from Sect. 3, we identified three groups, two of which were hope and non-hope; the last group (Other languages) was introduced to account for comments that were not in the required language. These classes were chosen since they provided a sufficient amount of generalisation for describing the remarks in the EDI hope speech dataset.

### Annotators

We created Google forms to collect annotations from annotators. To maintain the level of annotation, each form was limited to 100 comments and each page to ten comments. We collected information on the annotator’s gender, educational background and preferred medium of instruction in order to comprehend the annotator’s diversity and avoid bias. The annotators were warned that the comments may contain profanity and hostile material. If the annotator deemed the remarks to be too upsetting or unmanageable, they were offered the choice of ceasing to annotate. We trained annotators by directing them to YouTube videos on EDI.^6^$$^{,}$$^7^$$^{,}$$^8^$$^{,}$$^9^ Each form was annotated by at least three individuals. After the annotators marked the first form with 100 comments, the findings were manually validated in the warm-up phase. This strategy was utilised to help them acquire a better knowledge of EDI and focus on the project. Following the initial stage of annotating their first form, a few annotators withdrew from the project and their remarks were deleted. The annotators were told to conduct another evaluation of the EDI videos and annotation guidelines. From Table 1, we can see the statistics pertaining to the annotators. The annotators for English language remarks came from Australia, Ireland, the United Kingdom and the United States of America. We were able to obtain annotations in Tamil from persons from both India’s Tamil Nadu and Sri Lanka. Graduate and postgraduate students made up the majority of the annotators.

**Table 2:** Corpus statistic

Language pair	English	Tamil	Malayalam
Number of Words	522,717	191,242	122,917
Vocabulary Size	29,383	46,237	40,893
Number of Comments/Posts	28,424	17,715	9,817
Number of Sentences	46,974	22935	13,643
Average Number of Words per Sentence	18	9	11
Average Number of Sentences per Post	1	1	1

### Inter-annotator Agreement

We used the majority to aggregate the hope speech annotations from several annotators; the comments that did not get a majority in the first round were collected and added to a second Google form to allow more annotators to contribute them. We calculated the inter-annotator agreement following the last round of annotation. We quantified the clarity of the annotation and reported the inter-annotator agreement using Krippendorff’s alpha. Krippendorff’s alpha is a statistical measure of annotator agreement that indicates how well the resulting data corresponds to actual data [58]. Although **Krippendorff’s alpha** $(\alpha )$ is computationally hard, it was more relevant in our instance since the comments were annotated by more than two annotators and not all sentences were commented on by the same annotator. It is unaffected by missing data, allows for variation in sample sizes, categories and the number of raters and may be used with any measurement level, including nominal, ordinal, interval and ratio. $\alpha$ is characterised by the following:

1 $$\begin{aligned} \alpha = 1 - \frac{D_o}{D_e} \end{aligned}$$

$D_o$ is the observed disagreement between sentiment labels assigned by the annotators, and $D_e$ is the disagreement expected when the coding of sentiments can be attributed to chance rather than to the inherent property of the sentiment itself.

2 $$\begin{aligned} D_o= & {} \frac{1}{n}\sum _{c}\sum _{k}o_{ck\;metric}\;\delta ^2_{ck} \end{aligned}$$

3 $$\begin{aligned} D_e= & {} \frac{1}{n(n-1)} \sum _{c}\sum _{k}n_c \;.\;n_{k\;metric}\,\delta ^2_{ck} \end{aligned}$$

Here $o_{ck}\;n_c\;n_k\;$ and *n* refer to the frequencies of values in the coincidence matrices, and *metric* refers to any metric or level of measurement such as nominal, ordinal, interval, ratio and others. Krippendorff’s alpha applies to all these metrics. The range of $\alpha$ is between ‘0’ and ‘1’ and $1 \ge \alpha \ge 0$. When $\alpha$ is ‘1’, there is perfect agreement between the annotators, and when it is ‘0’, the agreement is entirely due to chance. It is customary to require $\alpha$ $\ge$.800. A reasonable rule of thumb that allows for tentative conclusions to be drawn requires $0.67 \le \alpha \le 0.8$ while $\alpha \ge$.653 is the lowest conceivable limit. For computing Krippendorff’s alpha (*alpha*) [59], we utilised *nltk*.^10^ Our annotations achieved agreement values of 0.63, 0.76 and 0.85 for English, Tamil and Malayalam, respectively, using the nominal measure. Previous research on sentiment analysis annotations and offensive language identification for Tamil and Malayalam in the code-switched settings achieved 0.69 for Tamil and 0.87 for Tamil in sentiment analysis as well as 0.74 for Tamil and 0.83 for Malayalam in offensive language identification. Our inter-annotator agreement (IAA) values for hope speech were close to the previous research on sentiment analysis and offensive language identification in Dravidian languages.

### Corpus statistics

Our dataset contains 59,354 YouTube comments, with 28,451 comments in English, 20,198 comments in Tamil and 10,705 comments in Malayalam. Our dataset also includes 59,354 comments in other languages. The distribution of our dataset is depicted in Table 2. When tokenising words and phrases in the comments, we used the nltk tool to obtain corpus statistics for use in research. Tamil and Malayalam have a broad vocabulary as a result of the various types of code-switching that take place.

Table 3 shows the distribution of the annotated dataset by the label in the reference tab: data distribution. As a result, the data was found to be biased, with nearly all of the comments being classified as ’not optimistic’ (NOT). An automatic detection system that can manage imbalanced data is essential for being really successful in the age of user-generated content on internet platforms, which is becoming increasingly popular. Using the fully annotated dataset, a train set, a development set and a test set were produced.

A few samples from the dataset, together with their translations and hope speech class annotations, are shown below.

- **kashtam thaan. irundhaalum muyarchi seivom** – *It is indeed difficult. Let us try it out though.* Hope speech
- **uff. China mon vannallo**– *Phew! Here comes the Chinese guy.* Non-hope speech
- **paambu kari saappittu namma uyirai vaanguranunga**– *These guys (Chinese) eat snake meat and make our lives miserable.* Non-hope speech

**Table 3:** Class-wise data distribution

Class	English	Tamil	Malayalam
Hope	2,484	7,899	2,052
Not Hope	25,940	9,816	7,765
Total	28,451	17,715	9,817

### Problematic examples

We found some problematic comments during the process of annotation.

- **’God gave us a choice.’** This sentence was interpreted by some as hopeful and others as not hopeful.
- ** Sri Lankan ** Tamilar history patti pesunga—*Please speak about history of Tamil people in Sri Lanka.* Inter-sentential switch in Tamil corpus written using Latin script. The history of Tamil people in Sri Lanka is both hopeful and non-hopeful due to the recent civil war.
- ** Bro helo app ku oru alternate appa solunga.**—* Bro tell me an alternate app for Helo app.* Intra-sentential and tag switch in Tamil corpus written using Latin script.

**Table 4:** Train-development-test data distribution

	English	Tamil	Malayalam
Training	22,735	13677	7,676
Development	2,843	2,018	1,070
Test	2,846	2,020	1,071
Total	28,424	17,715	9,817

**Table 5:** Precision, recall and F-score for English: support is the number of actual occurrences of the class in the specified dataset

Classifier	Hope Speech	Not-Hope Speech	Macro Avg	Weighted Avg
Support	250	2,593		
*Precision*				
SVM	0.23	0.91	0.30	0.83
MNB	0.24	0.91	0.35	0.84
KNN	0.63	0.92	0.52	0.90
DT	0.46	0.94	0.47	0.90
LR	0.33	0.96	0.43	0.90
RoBERTa	0.69	0.95	0.55	0.93
*Recall*				
SVM	0.22	1.00	0.33	0.83
MNB	0.19	1.00	0.33	0.91
KNN	0.14	0.99	0.38	0.92
DT	0.39	0.96	0.45	0.90
LR	0.59	0.88	0.49	0.86
RoBERTa	0.53	0.98	0.50	0.94
*F-score*				
SVM	0.21	0.95	0.32	0.87
MNB	0.20	0.95	0.31	0.87
KNN	0.23	0.96	0.40	0.89
DT	0.42	0.95	0.46	0.90
LR	0.43	0.92	0.45	0.87
RoBERTa	0.60	0.97	0.52	0.93

**Table 6:** Precision, recall and F-score for Tamil: support is the number of actual occurrences of the class in the specified dataset

Classifier	Hope Speech	Not-Hope Speech	Macro Avg	Weighted Avg
Support	815	946		
*Precision*				
SVM	0.00	0.47	0.16	0.22
MNB	0.58	0.57	0.63	0.60
KNN	0.48	0.55	0.53	0.52
DT	0.52	0.57	0.53	0.54
LR	0.59	0.59	0.55	0.58
RoBERTa	0.59	0.64	0.59	0.61
*Recall*				
SVM	0.00	1.00	0.33	0.47
MNB	0.42	0.81	0.49	0.58
KNN	0.35	0.72	0.48	0.53
DT	0.40	0.71	0.51	0.55
LR	0.37	0.73	0.58	0.57
RoBERTa	0.49	0.68	0.63	0.61
*F-score*				
SVM	0.00	0.64	0.21	0.30
MNB	0.49	0.67	0.51	0.56
KNN	0.41	0.62	0.49	0.51
DT	0.45	0.63	0.51	0.53
LR	0.46	0.65	0.55	0.56
RoBERTa	0.54	0.66	0.61	0.60

**Table 7:** Precision, recall and F-score for Malayalam: support is the number of actual occurrences of the class in the specified dataset

Classifier	Hope Speech	Not-Hope Speech	Macro Avg	Weighted Avg
Support	194	776		
*Precision*				
SVM	0.00	0.72	0.24	0.52
MNB	0.78	0.76	0.81	0.78
KNN	0.39	0.77	0.65	0.71
DT	0.51	0.81	0.61	0.73
LR	0.46	0.79	0.57	0.70
RoBERTa	0.70	0.91	0.81	0.87
*Recall*				
SVM	0.00	1.00	0.33	0.72
MNB	0.16	1.00	0.42	0.76
KNN	0.12	0.96	0.48	0.75
DT	0.27	0.92	0.53	0.76
LR	0.25	0.89	0.51	0.73
RoBERTa	0.72	0.91	0.81	0.87
*F-score*				
SVM	0.00	0.84	0.28	0.61
MNB	0.26	0.86	0.44	0.69
KNN	0.19	0.86	0.51	0.70
DT	0.36	0.86	0.56	0.73
LR	0.33	0.84	0.53	0.70
RoBERTa	0.71	0.91	0.81	0.87

## Benchmark experiments

We presented our dataset by utilising a broad range of common classifiers on the dataset’s imbalanced parameters, and the results were quite promising. The experiment was conducted on the token frequency-inverse document frequency (TF-IDF) relationship between tokens and documents. To generate baseline classifiers, we utilised the sklearn package (https://scikit-learn.org/stable/) from the sklearn project. Alpha = 0.7 was used for the multinomial Naive Bayes model. We employed a grid search for the k-nearest neighbours (KNN), a support vector machine (SVM), a decision tree and logistic regression. Detailed information on the parameters of the classifier will be made available in the code.

By using Facebook AI’s RoBERTa model, which is an upgraded version of the BERT model  [60], the company has achieved state-of-the-art results on numerous natural language understanding (NLU) tasks, including GLUE  [61] and SQUAD  [62]. RoBERTa is enhanced by training BERT for a longer period of time on longer sequences, increasing the amount of data available, eliminating the sentence prediction target during pre-training and modifying the masking pattern used during pre-training, among other things. It was created with the goal of increasing cross-lingual language understanding (XLU) by utilising a transformer-based masked language model, and it is known as XLM-RoBERTa (MLM). In order to train XLM-RoBERTa, it was fed 2 gigabytes of CommonCrawl data  [63], which had one hundred languages in total. It was found that XLM-RoBERTa surpasses its multilingual MLM competitors mBERT  [60] and XLM  [64] in terms of performance.

Using the training dataset, we trained our models; the development dataset was used to fine-tune the hyper-parameters, and the models were assessed by predicting labels for the held-out test set, as shown in Table 4. The performance of the categorisation was measured using a macro-averaged F-score, which was derived by averaging accuracy and recall over a large number of trials. Such a decision was made owing to the uneven class distribution, which causes well-known measures of performance such as accuracy and the micro-averaged F-score to be less than accurately representational of actual performance. Since the overall performance of all classes is important, we also presented the accuracy, recall and weighted F-score of the individual courses in addition to the overall performance. There are three tables in this section that provide the precision, recall and F-score findings of the HopeEDI test set employing baseline classifiers in conjunction with support from the test data: Table 5, Table 6 and Table 7.

As demonstrated, all of the models performed badly as a result of an issue with class imbalance. Using the HopeEDI dataset, the SVM classifier showed the worst performance, with macro-averaged F-scores of 0.32, 0.21 and 0.28 for English, Tamil and Malayalam, respectively. The decision tree led to a higher macro F-score for English and Malayalam than the logistic regression; however, Tamil fared well in both tests. In order to eliminate non-intended language comments from our dataset, we applied language identification techniques. The annotation ’Other languages’ was made in some comments by annotators although this was not the case in all of them. Another inconsistency was introduced into our dataset as a result of this. The majority of the macro scores were lower for English as a result of the ’Other languages’ category. In the case of English, this could have been prevented by simply eliminating those comments from the dataset. However, this label was required for Tamil and Malayalam since the comments in these languages were code-mixed and written in a script that was not native to the language (Latin script). The distribution of data for the Tamil language was roughly equal between the hope and non-hope classes.

The usefulness of our dataset was evaluated through the use of machine learning techniques, which we carried out in our trials. Due to its novel method of data collection and annotation, we believe the HopeEDI dataset has the potential to revolutionise the field of language technology. We believe that it will open up new directions in the future for further research on positivity.

## Task description

We also organised a shared task to invite more researchers to perform hope speech detection and benchmark the data. For our problem, we defined the hope speech as ’YouTube comments/posts that offer support, reassurance, suggestions, inspiration and insight’. A comment or post within the corpus may contain more than one sentence, but the average sentence length of the corpus is one. The annotations in the corpus were made at a comment/post level. The datasets for development, training and testing were supplied to the participants in English, Tamil and Malayalam.

### Training phase

During the first phase, participants were provided with training, validation and development data in order to train and develop hope speech detection for one or more of the three languages. Cross-validation on the training data was an option as was using the validation dataset for early evaluations and the development set for hyper-parameter sharing. The objective of this step was to guarantee that the participants’ systems were ready for review before the test data was released. In total, 137 people registered and downloaded the data in all three languages.

### Testing phase

The test dataset was provided without the gold labels in CodaLab during this phase. Participants were given Google forms to fill out in order to submit their predictions. They were given the option of submitting their findings as many times as they wished, with the best entry being picked for assessment and the creation of the rank list. The outcomes were compared to the gold standard labels. Across all classes, the classification system’s performance was assessed in terms of the weighted averaged precision, recall and F-score. The support-weighted mean per label was calculated using the weighted averaged scores. The metric used for preparing the rank list was the weighted F1 score. Participants were encouraged to check their systems using the Sklearn classification report.^11^ The final test included 30, 31 and 31 participants for Tamil, Malayalam and English languages, respectively.

## Systems

**Table 8:** Rank list based on F1-score along with other evaluation metrics (precision and recall) for the Tamil language

Team-Name	Precision	Recall	F1 Score	Rank
spartans [65]	0.62	0.62	0.61	1
TeamUNCC [66]	0.61	0.61	0.61	1
NLP@CUET [67]	0.61	0.61	0.6	2
res - si sun	0.61	0.6	0.6	2
team-hub [68]	0.61	0.61	0.59	3
MUCS [69]	0.59	0.59	0.59	3
ZYJ [70]	0.59	0.59	0.59	3
dhivya-hope-detection [71]	0.59	0.59	0.59	3
GCDH [72]	0.62	0.6	0.58	4
e8ijs	0.59	0.59	0.58	4
EDIOne - suman [73]	0.58	0.58	0.58	4
IIITK [74]	0.58	0.58	0.58	4
HopeIsAllYouNeed	0.59	0.59	0.57	5
IRNLP-DAIICT-LR [75]	0.59	0.59	0.57	5
KBCNMUJAL	0.59	0.59	0.57	5
KU-NLP [76]	0.62	0.6	0.56	6
Zeus [77]	0.59	0.59	0.56	6
CFILT-IITB-Submission	0.55	0.55	0.55	7
IIIT-DWD [78]	0.54	0.54	0.54	8
hopeful-nlp [79]	0.57	0.56	0.53	9
MUM	0.53	0.53	0.53	9
snehan-coursera	0.53	0.55	0.52	10
TeamX - Olawale Onabola	0.55	0.55	0.52	10
Hopeful-Men [80]	0.52	0.55	0.49	11
SIMON [81]	0.63	0.56	0.49	11
result	0.63	0.56	0.49	11
Amrita-CEN-NLP [82]	0.48	0.49	0.47	12
mIGeng	0.42	0.42	0.42	13
ssn-diBERTsity [83]	0.43	0.44	0.38	14
IIITT - Karthik Puranik [84]	0.38	0.39	0.37	15

**Table 9:** Rank list based on F1-score along with other evaluation metrics (precision and recall) for the Malayalam language

Team-Name	Precision	Recall	F1 Score	Rank
NLP@CUET[67]	0.86	0.85	0.85	1
MUCS [69]	0.85	0.85	0.85	1
GCDH [72]	0.84	0.85	0.85	1
ZYJ [70]	0.84	0.84	0.84	2
team-hub [68]	0.84	0.85	0.84	2
res - si sun	0.84	0.85	0.84	2
KU-NLP [76]	0.84	0.85	0.84	2
CFILT-IITB-Submission	0.84	0.85	0.84	2
TeamUNCC [66]	0.83	0.83	0.83	3
IIITK [74]	0.83	0.84	0.83	3
HopeIsAllYouNeed	0.83	0.83	0.83	3
EDIOne - suman t [73]	0.83	0.83	0.83	3
e8ijs	0.83	0.84	0.83	3
ssn-diBERTsity [83]	0.82	0.81	0.81	4
snehan-coursera	0.82	0.81	0.81	4
KBCNMUJAL	0.81	0.82	0.81	4
hopeful-nlp [79]	0.82	0.81	0.81	4
dhivya-hope-detection [71]	0.81	0.82	0.81	4
IIIT-DWD [78]	0.79	0.79	0.79	5
Zeus [77]	0.79	0.81	0.78	6
MUM	0.76	0.78	0.77	7
TeamX - Olawale Onabola	0.77	0.74	0.75	8
IRNLP-DAIICT-LR [75]	0.78	0.79	0.75	8
Hopeful-Men [80]	0.76	0.79	0.75	8
Amrita-CEN-NLP [82]	0.78	0.73	0.75	8
Amrita [82]	0.76	0.72	0.73	9
spartans [65]	0.62	0.62	0.61	10
mIGeng	0.58	0.61	0.59	11
IIITT - Karthik Puranik [84]	0.57	0.57	0.57	12
SIMON [81]	0.63	0.56	0.49	13
result	0.63	0.56	0.49	13

**Table 10:** Rank list based on F1 score along with other evaluation metrics (precision and recall) for the English language

Team-name	Precision	Recall	F1 score	Rank
Zeus [77]	0.93	0.94	0.93	1
TeamUNCC [66]	0.93	0.94	0.93	1
team-hub [68]	0.93	0.93	0.93	1
res - si sun	0.93	0.93	0.93	1
NLP@CUET[67]	0.93	0.93	0.93	1
KU-NLP [76]	0.92	0.93	0.93	1
Hopeful-men [80]	0.93	0.93	0.93	1
GCDH	0.93	0.93	0.93	1
EDIOne - suman t [73]	0.93	0.94	0.93	1
cs-english [85]	0.93	0.94	0.93	1
Autobots [86]	0.93	0.93	0.93	1
Hopeful-nlp [79]	0.93	0.94	0.93	1
ZYJ [70]	0.92	0.93	0.92	2
ssn-diBERTsity [83]	0.91	0.93	0.92	2
MUCS [69]	0.92	0.93	0.92	2
IRNLP-DAIICT-LR [75]	0.92	0.93	0.92	2
IIITK [74]	0.92	0.92	0.92	2
HopeIsAllYouNeed	0.92	0.93	0.92	2
dhivya-hope-detection [71]	0.92	0.92	0.92	2
CFILT-IITB-Submission	0.92	0.93	0.92	2
snehan-coursera	0.92	0.91	0.91	3
IIITT - Karthik Puranik [84]	0.92	0.91	0.91	3
MUM	0.89	0.91	0.9	4
IIIT-DWD [78]	0.9	0.91	0.9	4
e8ijs	0.91	0.92	0.9	4
wrecking-crew	0.9	0.91	0.87	5
HopeFighters	0.83	0.91	0.87	5
Amrita-CEN-NLP [82]	0.83	0.91	0.87	5
mlGeng	0.86	0.85	0.85	6
TeamX - Olawale Onabola	0.9	0.77	0.81	7
KBCNMUJAL	0.88	0.5	0.61	8

### System descriptions

In this section, we have summarised the systems implemented by the participants to complete the shared task. For more details, please refer to the shared task papers submitted by the authors.

- [71] participated in identifying hope speech classes in the English, Tamil and Malayalam datasets. They presented a two-phase mechanism to detect hope speech. In the first phase, they built a classifier to identify the language of the text. In the second phase, they created a classifier to identify the class labels. The author used the language models SBERT, FNN and BERT inference. They achieved the 3rd, 4th and 2nd ranks in Tamil, Malayalam and English, respectively.
- [76] used context-aware string embeddings for word representations and recurrent neural networks (RNNs) and pooled document embeddings for text representation. Their proposed methodology achieved a higher performance than the baseline results. The highest weighted average F-scores of 0.93, 0.56 and 0.84 for English, Tamil and Malayalam were reported on the final evaluation test set. The proposed models outperformed baselines by 3%, 2% and 11% in absolute terms for English, Tamil and Malayalam.
- [73] performed experiments by taking advantage of the pre-processing and transfer learning models. They showed that the pre-trained multilingual BERT model with convolution neural networks provided the best results. Their model ranked 1st, 3rd and 4th on the English, Malayalam-English and Tamil-English code-mixed datasets, respectively.
- [83] trained the data using transformer models, specifically mBERT for Tamil and Malayalam and BERT for English, and achieved weighted average F1 scores of 0.38, 0.81 and 0.92 for Tamil, Malayalam and English, respectively. They achieved the ranks of 14, 4 and 2 for Tamil, Malayalam and English, respectively.
- [84] experimented with several transformer-based models, including BERT, ALBERT, DistilBERT, XLM-RoBERTa and MuRIL, to classify the dataset into English, Malayalam and Tamil languages. ULMFiT achieved a weighted average F1 score of 0.91 on the English data, mBERT achieved 0.57 on the Malayalam data and distilmBERT achieved 0.37 on the Tamil data. They secured the 15th, 12th and 3rd ranks for predictions on the Tamil, Malayalam and English datasets, respectively.
- [78] used various machine learning- and deep learning-based models (SVM, logistic regression, convolutional neural network and RNN) to identify the hope speech in the given YouTube comments. The best-performing model on English data was 2-parallel CNN-LSTM with GloVe and Word2Vec embeddings, and it reported a weighted average F1 score of 0.91 and 0.90 for the development and test sets, respectively. Similarly, the best-performing model on Tamil and Malayalam data was obtained from 3-parallel Bi-LSTM. For Tamil, the reported F1 scores were 0.56 and 0.54 on the development and test datasets, respectively. Similarly, for Malayalam, the reported weighted F1 scores were 0.78 and 0.79 on the development and test datasets, respectively.
- [75] used TF-IDF character n-grams and pre-trained MuRIL embeddings for text representation as well as logistic regression and linear SVM for classification. Their best approach achieved the 2nd, 8th and 5th ranks with weighted F1 scores of 0.92, 0.75 and 0.57 in English, Malayalam-English and Tamil-English on the test dataset.
- [77] fine-tuned the RoBERTa pre-training model based on three datasets: English, Tamil and Malayalam. The F1 scores of their models in the Tamil and Malayalam sub-tasks reached 0.56 and 0.78, respectively, and the F1 score in the English sub-task reached 0.93, achieving the 1st rank.
- [70] used the attention mechanism to adjust the weights of all the output layers of XLM-RoBERTa to make full use of the information extracted from each layer, and they used the weighted sum of all the output layers to complete the classification task. They used the stratified k fold method to address class imbalance. They achieved weighted average F1 scores of 0.59, 0.84 and 0.92 for Tamil, Malayalam and English languages, which ranked 3rd, 2nd and 2nd, respectively.
- [68] used the method and model that combines the XLM-RoBERTa pre-raining language model and the TF-IDF algorithm. They secured the 1st, 2nd and 3rd ranks on the English, Malayalam and Tamil datasets, respectively.
- [85] used fine-tuned BERT and k fold cross-validation to accomplish classification on the English dataset. They achieved a final F1 score of 0.93 and secured the 1st rank for the English language.
- [72] demonstrated that even very simple baseline algorithms perform reasonably well in this task if provided with enough training data. However, their best-performing algorithm was a cross-lingual transfer learning approach where they fine-tuned XLM-RoBERTa. The model achieved the 1st rank for Malayalam and English and the 4th rank for Tamil.
- [66], in their paper, described their approach of fine-tuning RoBERTa for hope speech detection in English and fine-tuning XLM-RoBERTa for hope speech detection in the Tamil and Malayalam languages. They ranked 1st in English (F1 = 0.93), 1st in Tamil (F1 = 0.61) and 3rd in Malayalam (F1 = 0.83).
- [86] described a transformer-based BERT model for hope speech detection. Their model achieved a weighted averaged F1 score of 0.93 on the test set for English. They showed that the BERT model helped in providing better contextual representation of words in a comment and that the language identification model assisted in detecting comments in the ‘Other languages’ category. They also explored the use of other transformer models such as RoBERTa, XLNet, Albert, FLAIR and ELMo for a superior hope speech detection.
- [82] proposed a BiLSTM with an attention-based approach to solving hope speech detection, and using this approach, they achieved an F1 score of 0.73 (9th rank) in the Malayalam–English dataset.
- [80] experimented with two approaches. In the first approach, they used contextual embeddings to train classifiers using logistic regression-, random forest-, SVM- and LSTM-based models. The second approach involved using a majority voting ensemble of 11 models that were obtained by fine-tuning pre-trained transformer models (BERT, AL-BERT, RoBERTa and IndicBERT) after adding an output layer. They found that the second approach was superior for English, Tamil and Malayalam. They got a weighted F1 score of 0.93, 0.75 and 0.49 for English, Malayalam and Tamil, respectively. They ranked 1st in English, 8th in Malayalam and 11th in Tamil.
- [79] achieved an F-score of 0.93, ranking 1st on the leaderboard for English comments. The paper used pre-trained transformers and Paraphrasing Generation for Data Augmentation.
- [67] employed various machine learning (SVM, LR and ensemble), deep learning (CNN + BiLSTM) and transformer-based (m-BERT, Indic-BERT, XLNet and XLM-R) methods. They showed that XLM-R outperformed all other techniques by gaining a weighted F1 score of 0.93, 0.60 and 0.85, respectively, for the English, Tamil and Malayalam languages. Their team achieved the 1st, 2nd and 1st ranks in these three tasks, respectively.
- [81] used the XLM- RoBERTa model and proposed an excellent multilingual model to complete the classification task.
- [69] created three models, namely CoHope-ML, CoHope-NN and CoHope-TL based on Ensemble of classifiers, the Keras neural network (NN) and BiLSTM with the Conv1D model. The CoHope-ML and CoHope-NN models were trained on a feature set comprising char sequences extracted from sentences combined with words for Malayalam–English and Tamil-English code-mixed text and a combination of word and char n-grams along with syntactic word n-grams for English text. The CoHope-TL model consisted of three major parts: training tokeniser, BERT language model (LM) training and then using the pre-trained BERT LM as weights in the BiLSTM - Conv1d model. Out of the three proposed models, the CoHope-ML model (the best one among the models proposed) obtained the 1st, 2nd and 3rd ranks with weighted F1 scores of 0.85, 0.92 and 0.59 for Malayalam-English, English and Tamil-English texts, respectively.
- [65] extended the work of Arora (2020a), as they used their strategy to synthetically generate code-mixed data for training a transformer-based RoBERTa model and used it in an ensemble along with their pre-trained ULMFiT. They presented the RoBERTa language model for code-mixed Tamil, which they had pre-trained from scratch. Using transfer learning, they fine-tuned the RoBERTa and ULMFiT language models on downstream tasks of OLI and HSD. They secured the 4th rank in the former task using an ensemble of classifiers trained on RoBERTa and ULMFiT and the 1st rank in the latter task using the classifier based on ULMFiT.

## Results and discussion

Overall, we received a total of 31, 31 and 30 submissions for English, Malayalam and Tamil tasks. It is interesting to note that the top-performing teams in all the three languages predominantly used XLM-RoBERTa to complete the shared task. One of the top-ranking teams for English used context-aware string embeddings for word representations and RNNs as well as pooled document embeddings for text representation. Among the other submissions, although Bi-LSTM was popular, there were other machine learning and deep learning models that were used. However, they did not achieve good results compared to the RoBERTa-based models.

The top scores were 0.61, 0.85 and 0.93 for Tamil, Malayalam and English, respectively. The ranges of scores were between 0.37 and 0.61, 0.49 and 0.85 and 0.61 and 0.93 for the Tamil, Malayalam and English datasets, respectively. It can be seen that the F1 scores of all the submissions on the Tamil dataset were considerably lower than those of Malayalam and English. It is not surprising that the English scores were better, as many approaches used variations of pre-trained transformer-based models trained on English data. Due to code-mixing at various levels, the scores were naturally lower for the Malayalam and Tamil datasets. Among these two, the systems submitted performed badly on Tamil data. The identification of the exact reasons for the bad performance in Tamil requires further research. However, one possible explanation for this could be that the distribution of the ’Hope_speech’ and ’Non_hope_speech’ classes in Tamil was starkly different from that in English and Malayalam. In the remaining two classes, the number of non-hope speech comments were significantly higher than hope speech comments.

## Conclusion

As online content increases massively, it is necessary to encourage positivity, such as in the form of hope speech on online forums, to induce compassion and acceptable social behaviour. In this paper, we presented the largest manually annotated dataset of hope speech detection in English, Tamil and Malayalam consisting of 28,451, 20,198 and 10,705 comments, respectively. We believe that this dataset will facilitate future research on encouraging positivity. We aimed to promote research on hope speech and encourage positive content on online social media for ensuring EDI. In the future, we plan to extend the study by introducing a larger dataset with further fine-grained classification and content analysis.

## Data Availability

The datasets used in this paper were obtained from https://huggingface.co/datasets/hope_edi.

## Footnotes

1 http://www.terpconnect.umd.edu/~jklumpp/ARD/MilkSpeech.pdf.

2 https://www.youtube.com/.

3 https://github.com/philbot9/youtube-comment-scraper.

4 We considered women in the fields of STEM, people belonging to the LGBTQIA+ community, racial minorities or people with disabilities as a part of the EDI community for this study.

5 https://pypi.org/project/langdetect/.

6 https://www.youtube.com/watch?v=C-uyB5I6WnQ &t=6s.

7 https://www.youtube.com/watch?v=UcuS5glhNto.

8 https://www.youtube.com/watch?v=hNeR4bBUj68.

9 https://www.youtube.com/watch?v=LqP6iU3g2eE.

10 https://www.nltk.org/.

11 https://scikit-learn.org/stable/modules/generated/sklearn.metrics.classification_report.html.
